# Do Cenobamate Pharmacokinetics Change with Co-Administered Antiseizure Medications? An Exploratory Analysis of Responder Patients with Focal Drug-Resistant Epilepsy

**DOI:** 10.3390/pharmaceutics18010092

**Published:** 2026-01-10

**Authors:** Bruno Charlier, Viviana Izzo, Giovanni Assenza, Anna Chiara Balsamo, Flavia Cirillo, Albino Coglianese, Carlo Di Bonaventura, Mariana Fernandes, Antonio Gambardella, Emanuele Cerulli Irelli, Claudio Liguori, Sandra Rufolo, Ilaria Sammarra, Amelia Filippelli, Francesca Felicia Operto

**Affiliations:** 1 Operative Unit of Clinical Pharmacology, University Hospital “San Giovanni di Dio e Ruggi d’Aragona”, 84131 Salerno, Italy; vizzo@unisa.it (V.I.);; 2 Post-Graduate School in Clinical Pharmacology and Toxicology, University of Salerno, Baronissi, 84081 Salerno, Italy; 3 Department of Medicine, Surgery and Dentistry “Scuola Medica Salernitana”, University of Salerno, 84081 Salerno, Italy; 4 Research Unit of Neurology, Department of Medicine and Surgery, Università Campus Bio-Medico di Roma, Via Alvaro del Portillo, 21, 00128 Roma, Italy; 5 Operative Research Unit of Neurology, Fondazione Policlinico Universitario Campus Bio-Medico, Via Alvaro del Portillo, 200, 00128 Roma, Italy; 6 Department of Systems Medicine, University of Rome Tor Vergata, 00133 Rome, Italy; 7 Neurology Unit, University Hospital of Rome Tor Vergata, 00133 Rome, Italy; 8 Post-Graduate School in Clinical Pathology and Clinical Biochemistry, University of Salerno, Baronissi, 84081 Salerno, Italy; 9 Department of Human Neurosciences, Sapienza University, 00185 Rome, Italy; 10 Department of Science of Health, School of Medicine, Magna Græcia University, 88100 Catanzaro, Italy; 11 Neurologic Clinic, Magna Græcia University, 88100 Catanzaro, Italy; 12 Department of Medical and Surgical Sciences, Institute of Neurology, Magna Græcia University, 88100 Catanzaro, Italy; 13 Centre for Research in Unusual Infections, Epilepsy and Neuroscience (CRUISE), Science of Health Department, Magna Græcia University, 88100 Catanzaro, Italy

**Keywords:** cenobamate, antiseizure medication, pharmacokinetics, therapeutic drug monitoring

## Abstract

**Background:** Cenobamate (CNB) is an anti-seizure medication (ASM) approved for the treatment of drug-resistant focal epilepsy in adults. Notwithstanding significant proof of efficacy, real-world pharmacokinetics (PK) data are lacking, particularly regarding sex-based variations and the effect of concomitant ASMs. This exploratory study aimed to investigate the PK profile of CNB in responder adults with drug-resistant focal epilepsy and assess potential relationship with concomitant ASMs and clinical variables. **Methods:** We enrolled 17 patients receiving add-on CNB. The concentration-to-dose ratio (C/D), incremental slope (ΔC/ΔD), and dose-to-concentration AUC were calculated. Enrolled individuals were stratified into three exposure clusters (low, medium, and high). Univariate ANOVA was used to explore associations between PK parameters, clinical variables and concomitant ASMs. **Results:** Sex appeared to be associated with AUC cluster classification (*p* = 0.026), showing females predominating in the high-exposure group. A nonlinear dose-concentration relationship emerged from the ΔC/ΔD analysis, showing steeper slopes at low doses (12.5–50 mg), great variability at higher doses (100–200 mg), and a negative slope in some individuals. Higher CNB concentrations were observed in patients co-treated with lacosamide, while concomitant topiramate was associated with lower exposure. Carbamazepine and valproate showed non-significant trends consistent with their known enzyme-inducing and inhibiting properties. **Conclusions:** PK of CNB appears highly variable and seems to be influenced by sex and concomitant ASMs. These findings highlight the importance of therapeutic drug monitoring and individualized titration strategies to optimize efficacy and safety in clinical practice. These results should be regarded as exploratory and hypothesis-generating due to the small and monocentric sample size and need to be confirmed in larger, multicenter cohorts.

## 1. Introduction

Cenobamate (CNB) has been approved by the U.S. Food and Drug Administration (FDA) as an adjunctive therapy for adults with drug-resistant focal epilepsy [1]. Based on therapeutic outcomes, CNB treatment allows add-on or monotherapy use. This may enable dose reduction [2,3] or withdrawal of other antiseizure medications (ASMs) [4]. Although CNB pharmacokinetics (PK) and mechanism of action are partially known [5,6,7,8], there is still a lack of data about its kinetic profile, particularly in polytherapy regimens [9,10]. In clinical practice, treatment adjustments are often made empirically, based only on the therapeutic response. This can frequently lead to a reduction or discontinuation of other ASMs (e.g., sodium channel blockers, benzodiazepines) without considering their PK interactions or plasma concentrations. Indeed, PK modeling studies have proven that concomitant use of clobazam (CLB) or carbamazepine (CBZ) significantly affects CNB clearance [11].

In humans, the intra-individual variability of CNB has been estimated to be as high as 14% for Cmax and 5% for AUCt, while the inter-individual variability may reach 25% for Cmax and 35% for AUCt [1]. In vitro studies suggest that CNB has the potential to both induce and inhibit cytochrome P450 isoenzymes, specifically CYP2B6 and CYP3A4, as well as inhibit CYP2C19 and induce CYP2C8 [12]. Phase I trials have shown complex bidirectional interactions between CNB and other antiepileptic medications (ASMs), including carbamazepine (CBZ), phenytoin (PHT), and phenobarbital (PB) [13]. In more detail, CNB increased PHT and PB plasma exposure by 84% and 37%, respectively, likely inhibiting CYP2C19. Conversely, PHT and PB reduced CNB AUC by 28% and 15%, respectively [14]. Nonlinear PK has also been observed with increasing CNB doses: oral clearance decreases by about 70% when CNB dosage rises above 200 mg (from 1.4 to 0.4 L/h), suggesting nonlinear distribution and/or elimination processes [6]. Nevertheless, most of the current evidence is based on phase I studies or large heterogeneous cohorts, which may not reflect real-world clinical populations characterized by polytherapy and various demographic profiles [15]. According to available data, CNB can substantially influence the metabolism of several ASMs. However, because multiple enzymatic systems are involved, often with compensatory mechanisms, their clearance and overall PK remain difficult to predict [16].

The present study is part of a multicenter investigation called “A multicenter, ambispective study to evaluate the pharmacological exposure to CNB in patients with epilepsy,” carried out in accordance with the Declaration of Helsinki and the International Conference on Harmonization of Good Clinical Practices (ICH-GCP) guidelines [17,18]. This exploratory study aimed to investigate the PK profile of CNB in responder adults with drug-resistant focal epilepsy with add-on CNB and to assess PK potential change in relation to concomitant ASMs and clinical variables. Complete PK profiles were obtained at each dose titration step [19]. The study was focused on 17 responder individuals enrolled from a single center, thereby ensuring consistent titration procedures and comparable CNB exposure levels. We examined the relationships between PK parameters (AUC, ΔC/ΔD slope and concentration/dose ratio), concomitant ASMs and clinical variables (sex, age, BMI and disease duration). Our findings indicate that PK profile of CNB is highly variable and appears to be influenced by sex and concomitant ASMs. These results highlight the importance of therapeutic drug monitoring and personalized titration regimens in clinical practice to improve CNB efficacy and safety.

## 2. Materials and Methods

### 2.1. Study Design and Participant Enrollment

Data from 17 patients enrolled at the Operational Unit of Neuropsychiatry of the Department of Health Sciences at the University of Catanzaro “Magna Graecia” (Italy) were analyzed. Participants were selected according to previously described inclusion and exclusion criteria. Details of patient selection and titration schedules have been extensively reported elsewhere [19]. For each participant, anthropometric and clinical data were collected, including age, sex, weight, BMI, age at onset, epilepsy duration, number of monthly seizures at baseline, comorbidities and details of medications taken during the titration phase (including ASMs and other medications). To ensure treatment homogeneity, only individuals with focal epilepsy who responded to add-on CNB therapy were included in the analysis. A subgroup of 17 responders from a larger multicenter cohort was included in the presented PK analysis since they were enrolled in the same center and had CNB concentration data for every titration step. Non-responders could not be consistently included in this PK evaluation, as they were often stopped CNB prematurely or had insufficient sampling points. Because they were not routinely collected, body fat percentage, free (unbound) CNB values, and comprehensive hepatic/renal biomarker panels could not be included as variables in pharmacokinetic analyses.

### 2.2. Sample Collection and Analysis

Whole-blood samples were collected at baseline and at each dose escalation step using BD Vacutainer^®^ tubes containing 2,2′,2″,2‴-(ethane-1,2-diyldinitrilo) tetraacetic acid (EDTA) as an anticoagulant. Samples were centrifuged at 3500× *g* for 6 min, and the resulting plasma was transferred into clean safe-lock tubes and stored at −20 °C until analysis. Blood samples for CNB measurement were collected during routine outpatient visits at each titration step, when the relative dosage steady state had presumably reached. CNB plasma concentrations (CNBp) were quantified using a validated ultra-high-performance liquid chromatography coupled with tandem mass spectrometry (UHPLC-MS/MS) method, as described by Charlier and coworkers [20,21], and validated according to EMA/FDA bioanalytical method guidelines [22]. LC-MS/MS analyses were carried out on a Waters Xevo TQ-XS triple quadrupole mass spectrometer coupled to a Waters Acquity UPLC I-Class System (Waters Corporation, Sesto San Giovanni, Italy). Ultra-pure solvents and formic acid were purchased from Romil (Waterbeach, Cambridge, UK); CNB (100 mg) was purchased from DC Chemicals^®^ (Shanghai, China). The deuterated IS lamotrigine-13C3-d3 (1 mg) was obtained from LGC standards (Milan, Italy).

### 2.3. Pharmacokinetic Parameters

At each titration step, PK parameters of CNB were calculated based on plasma concentration. The concentration-to-dose (C/D) ratio was obtained by dividing the CNBp (µg/mL) by the corresponding daily dose (mg/day). The incremental slope (ΔC/ΔD) was derived from the change in concentration (ΔC) relative to the change in dose (ΔD) between two consecutive titration steps, providing an estimate of dose-concentration linearity.

The trapezoidal approach was used to calculate the area under the dose-concentration curve (AUC), which included graphing the CNB dose on the *x*-axis (0 to 200 mg) and the measured plasma concentration on the *y*-axis at each titration step. CNB concentration was integrated over the range of administered doses to describe the overall drug exposure. Based on AUC values, participants were stratified into exposure clusters to enable comparison with clinical and pharmacological variables. Patients were divided into three exposure clusters (low AUC < 1400 mg/mL, medium 1400 < AUC < 2300 mg/mL, and high AUC > 2300 mg/mL) based on AUC distribution tertiles.

### 2.4. Data Collection and Statistical Analysis

Clinical information was retrieved from electronic medical records. Data on concomitant ASM dosages, seizure frequency, biometric parameters and follow-up interviews were collected at each visit. Both parametric and nonparametric tests were used in the statistical analysis, according to data distribution. Analysis of variance (ANOVA) and Fisher or Chi^2^ tests were used to assess associations between AUC cluster and clinical/pharmacological variables. Effect size was estimated using Cohen’s d. Fisher’s *t*-test, Mann–Whitney U, Pearson/Spearman correlations, as well as logistic and linear regression analyses, were used to evaluate the relationship between variables. To control for potential false positives due to small sample size, false discovery rate (FDR) correction was applied using the Benjamini–Hochberg method in independent blocks. Where applicable, effect sizes (Cohen’s d, Cliff’s delta) were also provided. Both unadjusted and FDR-corrected *p*-values, together with effect sizes, are available as Supplementary Materials in Table S1. The robustness of the observed connections between CNB exposure and concurrent ASMs was further evaluated by testing exploratory linear models that included CNB dose, sex and BMI as factors. These adjusted analyses were only regarded as hypothesis-generating due to the small sample size. All analyses were performed using the R statistical program (v4.1.2; R Core Team 2021).

### 2.5. Ethical Implications

This is part of an ambispective observational project based on clinical data that was approved by the local ethics committee. All participants provided written informed consent at the time of enrollment. The study was approved by the Ethics Committee of the “Magna Grecia” University (Registry Protocol No. 101 of 28 March 2024 of the TERRITORIAL ETHICS COMMITTEE OF THE CALABRIA REGION). Established with DDR No. 7927 OF 7 June 2023, Department of Health and Welfare 1) of Catanzaro (Italy) and conducted in accordance with the principles of the Declaration of Helsinki [18].

## 3. Results

### 3.1. Clinical Features

The study population included 17 adults with drug-resistant focal epilepsy, with a slight female prevalence (52.9%). Anthropometric measurements showed a distribution between normal weight and overweight, with a mean BMI of 25.44 ± 4.34 kg/m^2^. Clinical and demographic features of the study population are provided in Table 1.

### 3.2. Pharmacokinetic-Derived Parameters

**Figure 1:**
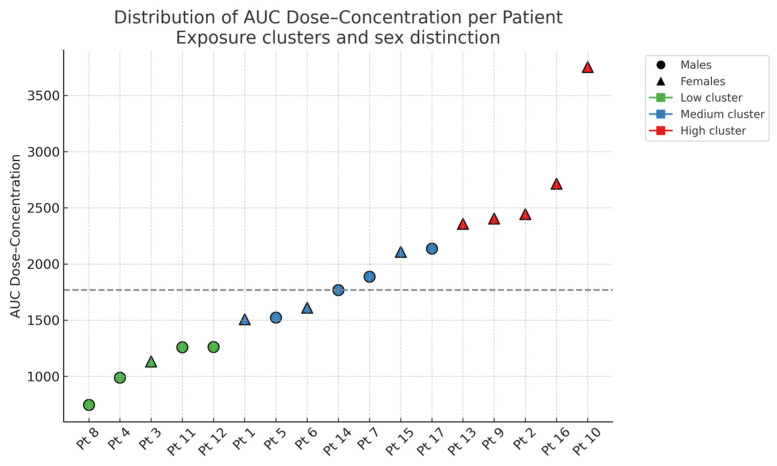
Distribution of CNB dose-concentration AUC values for each enrolled individual, stratified by exposure cluster and sex. The dashed horizontal line indicates the cohort’s median AUC value.

For each enrolled individual, CNB pharmacokinetic parameters (AUC, ΔC/ΔD and C/D ratio) were obtained from plasma concentration data collected across titration steps. Based on AUC values, participants were stratified into three exposure clusters (Low, Medium and High). A significant association was observed between sex and AUC cluster (*p* = 0.026, Fisher’s exact test), with a higher proportion of women in the high-exposure group (Figure 1). Although residual confounding cannot be definitively excluded, there was no systematic difference in the administered CNB maintenance dose between males and females in this small cohort, indicating that dose alone is unlikely to entirely account for the higher exposure cluster in women. As shown, AUC values showed wide interindividual variability, from approximately 700 to over 3700. No significant differences were found across clusters for age, BMI or epilepsy duration.

To quantify the change in plasma concentration as a function of the administered dose, we calculated the incremental slope (ΔC/ΔD) between pairs of successive titration steps using the following equation:

$$Slope=\frac{\Delta C}{\Delta D}=\frac{C_{i+1}-C_{i}}{{Dose}_{i+1}-{Dose}_{i}}$$

where

ΔC = absolute change in CNBp between two consecutive titration steps

ΔD = corresponding dose increment

C_i_ = CNBp at the dosage level Dose i

C_i+1_ = CNBp at the subsequent dosage level Dose i + 1

Dose_i_ = CNB dose administered at titration step i (mg/day)

Dose_i+1_ = CNB dose administered at the next titration step (i + 1)

**Figure 2:**
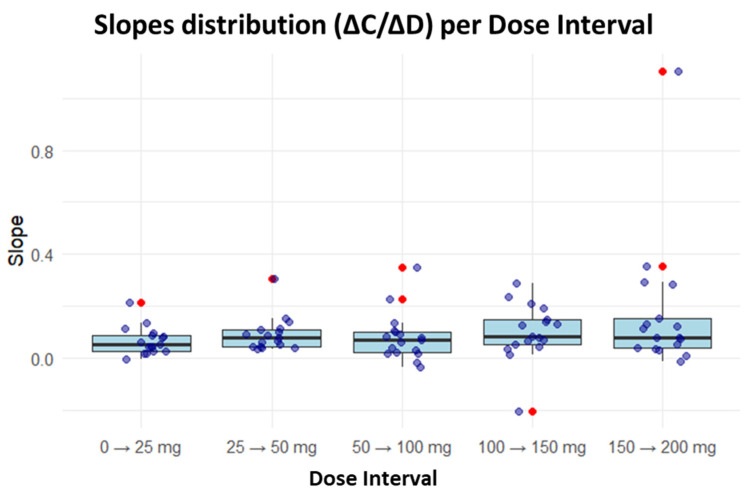
Distribution of incremental slopes across CNB titration intervals. For each interval, a boxplot is displayed showing the median (central line), the first and third quartiles (box limits), and whiskers extending to the extreme values without considering outliers. Individual observed values are represented as points overlaid on the boxplot, with outliers highlighted in red—these are anomalous values that deviate significantly from the main distribution, defined as observations falling beyond 1.5 times the interquartile range from their respective quartiles. The arrow indicates the transition between successive dose escalation steps.

Analysis of the derived parameters revealed a nonlinear dose–concentration relationship. In the first three titration intervals (0–25 mg, 25–50 mg and 50–100 mg), the median slope (ΔC/ΔD) remained relatively constant. However, in the final two intervals (100–150 mg and 150–200 mg), greater dispersion (wider IQR) and several outliers were observed. Occasionally, negative slopes were identified, indicating a decrease in CNBp despite dose escalation. Most slope values, representing the typical PK range, were between 0.05 and 0.15 µg/mL/mg (Figure 2).

The anthropometric (sex, age, weight, BMI) and clinical variables (age of onset, epilepsy duration) were compared across PK exposure clusters. Age was classified into three groups: <40, 40–60 and >60. The corresponding data are summarized in Table 2. AUC values ranged from 745 to 3754, reflecting substantial interindividual variability in CNB exposure. No consistent trends were observed among clusters for age, BMI, or epilepsy duration.

**Figure 3:**
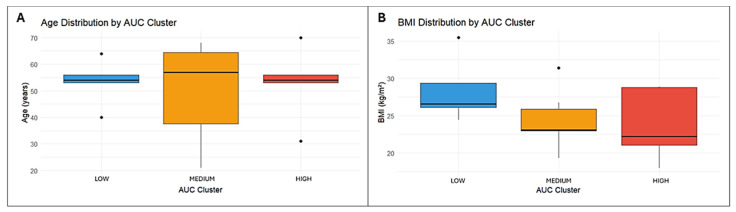
Distribution of age (**A**) and BMI (**B**) across AUC exposure clusters. Boxes indicate the interquartile range with the median, whiskers show the data range, and dots represent outliers.

One-way ANOVA was performed to compare AUC clusters with anthropometric and clinical variables. Although mean BMI values slightly differed among clusters, the difference was not statistically significant (*p* = 0.213). Similarly, age showed greater dispersion within the medium-exposure cluster, while the low- and high-exposure groups displayed more uniform distributions. However, this variability did not reach statistical significance (*p* = 0.915), indicating no association between age and CNB exposure. All results are illustrated in Figure 3.

A statistically significant association was found between sex distribution and AUC clusters (*p* = 0.026). Sex was coded as a binary variable (Female = 0, Male = 1). The marked differences in cluster composition suggest that PK exposure to CNB may be influenced by sex, with females more represented in the high-exposure group and males more frequent in the low- and medium-exposure clusters (Table 3).

### 3.3. Influence of Concomitant ASMs on CNB Response

The potential association between AUC exposure clusters and concomitant ASMs was evaluated. Fisher’s exact test and the chi-square test, when applicable, were used for the analysis. Concomitant ASMs were coded as binary categorical variables (0 = absent, 1 = present). The medications included levetiracetam (LEV) [4,23], perampanel (PER), valproic acid (VPA), eslicarbazepine (ESL), lamotrigine (LTG), CBZ, lacosamide (LCM), brivaracetam (BRV), PB [13], oxcarbazepine (OXC), gabapentin (GBP), CLB, cannabidiol (CBD), and topiramate (TPM). Each ASM was analyzed independently, using CNBp as the continuous dependent variable. Overall, no statistically significant association was observed between any concomitant ASM and AUC clusters. Fisher’s exact test *p*-value for LCM was near the significance threshold (*p* = 0.0609), suggesting a potential trend that warrants further investigations. The distribution of the other ASMs, including the most frequently used agents, such as CBZ, VPA, and LEV, did not differ significantly among exposure groups. Univariate analyses have shown that concomitant LCM was associated with higher CNB exposure, while concomitant TPM was associated with lower exposure (*p* < 0.05). Although not statistically significant in this sample, CBZ and VPA showed trends towards lower and higher concentrations, respectively, consistent with their known enzyme-inducing and -inhibiting properties (Table 4).

Given the limited sample size, these findings were further explored using effect size estimation (Cohen’s d) and false discovery rate (FDR) correction. After FDR adjustment and inclusion of exploratory covariates (CNB dose, sex and BMI), none of the observed associations remained statistically significant, and effect sizes were small. Therefore, rather than representing conclusive evidence of metabolic drug–drug interactions, these findings should be interpreted as exploratory patterns requiring confirmation in larger and independent cohorts (Supplementary Table S1).

## 4. Discussion

Our findings highlight the complexity of the PK profile of CNB, characterized by marked interindividual variability and non-linearity. Given the observational design and limited sample size, these results should be considered exploratory and hypothesis-generating. Sex stratification revealed that females were more prevalent in the high-exposure cluster, suggesting possible sex-related PK differences, consistent with observations reported for other ASMs. Sex-dependent variability in ASM pharmacokinetics has already been hypothesized and should probably be attributed to alterations in body composition, hormonal status, hepatic enzyme activity and protein binding. A potential sex-related impact on CNB pharmacokinetics is indicated by the overrepresentation of females in the high-exposure cluster. Nevertheless, there was no discernible dose imbalance between the sexes, and we lacked access to body fat percentage, free CNB fraction, or specific hepatic and renal biomarkers. Therefore, rather than representing a definitive mechanistic explanation, this finding should be interpreted as an exploratory signal consistent with the varied and occasionally contradictory information on sex-related changes in ASM pharmacokinetics. VPA and LTG, for example, have been shown to have such effects [24]. Variability increased at higher doses, where dose increments did not always correspond to proportional changes in CNBp. In some individuals, negative ΔC/ΔD slopes may reflect drug interactions, metabolic saturation, non-compliance or analytical variability, whereas the steeper slopes observed between 100 and 200 mg might indicate threshold-related changes in absorption or metabolism. Studies on dose escalation in healthy volunteers have revealed less-than-proportional increases in CNB exposure; at higher doses, mean apparent oral clearance (CL/F) drops from about 1.4 L/h to 0.4 L/h. At higher doses, the pharmacokinetic deviation from dose-proportionality may be caused by several real-world variables, such as non-standardized sampling times, variations in titration schedules, inter-individual differences in adherence and potential differences in bioavailability or partial saturation of first-pass metabolism. Due to these constraints, the nonlinearity should be interpreted cautiously because our data cannot differentiate among the different causes. Rather than full enzyme saturation, this deviation from dose proportionality points to either partial first-pass metabolism saturation or alterations in bioavailability [6]. “Classic” ASMs like PHT and VPA have also been shown to have similar non-linear kinetics, with capacity-limited metabolism explaining disproportionate concentration rises at higher doses [25].

Outliers may reflect pharmacogenetics or interindividual variability in absorption/distribution. Further studies integrating pharmacogenetics analysis could help clarify these sources of variability and support population PK modeling. No clear relationship was found between CNB exposure and age, BMI or epilepsy duration, suggesting that demographic or disease-related factors may play a minor role compared with metabolic or drug-interaction mechanisms.

The non-linear relationship between CNB dose and plasma concentration observed in this study suggests a deviation from dose-proportional pharmacokinetics at higher doses. In the study design, we explained the choice to include only responder patients with complete pharmacokinetic profiles; thus, the data presented in this work cannot capture potential pharmacokinetic differences between responder and non-responder patients and provides an exploratory overview that may not cover the full range of CNB exposure observed in clinical practice. In a dose-proportional (linear) model, plasma concentrations increase in direct proportion to the administered dose. However, when the metabolic or elimination pathways become saturated, this relationship may shift to a capacity-limited behavior, where additional dose increments produce smaller or inconsistent changes in plasma levels. Such non-linearity may arise from partial saturation of CNB metabolic enzymes (e.g., CYP2C19 or CYP3A4), competitive interactions with concomitant ASMs, or interindividual variability in hepatic clearance and protein binding. These mechanisms could account for the greater dispersion in ΔC/ΔD values and the occasional decrease in concentration despite dose escalation. From a clinical perspective, this variability reinforces the need for individualized titration and close pharmacokinetic monitoring, particularly at higher doses where concentration–response relationships become unpredictable. It has been repeatedly demonstrated that therapeutic drug monitoring minimizes interindividual variability and maximizes ASM treatment, especially for medications with complex pharmacokinetics or narrow therapeutic indices [26,27]. Pending additional validation, the application of therapeutic drug monitoring to CNB may prove beneficial during the titration or polytherapy stages. The observed associations with certain concomitant ASMs suggest potential PK interactions. In particular, individuals co-treated with TPM tended to show lower CNB concentrations, while those receiving LCM exhibited unexpectedly higher levels. These differences may reflect indirect metabolic interactions, modulation of hepatic transporters or specific characteristics of the treated population. In our cohort, TPM doses were typically reduced during CNB titration, whereas LCS doses were increased, findings consistent with previous clinical observations [19]. This study shows several limitations. The most relevant is the small sample size, which restricts the statistical power and generalizability of the findings. The weak association between TPM and LCM with CNB exposure could be partially explained by pharmacokinetic variability unrelated to direct metabolic modulation or dosage modifications made during titration. TPM doses were frequently lowered while LCM doses rose among responders in our real-world sample, which may have added confounding factors as a result of therapy modification and indication. These findings should be considered exploratory and require validation in larger studies due to the lack of statistical significance following FDR correction, as well as the previously indicated limitations of this investigation, including the chosen responder-only cohort. Moreover, the observational design and the absence of a controlled dosing protocol may have introduced variability related to clinical management and concomitant therapies. For these reasons, the present work should be considered exploratory in nature, aimed at generating hypotheses and guiding future pharmacokinetic investigations on CNB in real-world settings. The small sample size (n = 17), which is limited to responders and comes from a single center, significantly lowers the statistical power and raises the possibility of Type I and Type II errors. As a result, all correlations found in this study should be viewed cautiously as preliminary and hypothesis-generating and need to be confirmed in a larger and, ideally, multicenter cohort.

## 5. Conclusions

This exploratory study suggests that PK profiles of CNB are highly variable and may be influenced by sex and concomitant therapies. Preliminary signals emerged for LCM and TPM, while CBZ and VPA showed non-significant trends consistent with their known enzymatic profiles. The unexpected TPM effect warrants further investigation in larger cohorts. The observed non-linearity at higher doses further emphasizes the PK complexity of CNB and the potential role of metabolic saturation and interindividual variability. Our findings highlight the clinical relevance of therapeutic drug monitoring and individualized titration strategies, particularly in patients receiving polytherapy or presenting clinical and demographic features associated with variable CNB exposure. Given the small sample size, these results should be considered preliminary and hypothesis-generating, requiring confirmation in prospective multicenter studies. Although our study focused on pharmacokinetic variability, we did not attempt to define CNB therapeutic ranges or concentration–response thresholds. To date, no universally accepted therapeutic window has been established for CNB, and available data on exposure–response relationships remain limited. In our small responder-only cohort, any attempt to derive such thresholds would be highly biased and methodologically unsound; thus, our results should not be interpreted as defining target concentrations for seizure control. In conclusion, this small, single-center cohort composed exclusively of responder patients confirms the high interindividual variability and non-linearity of CNB pharmacokinetics and suggests potential influences of sex and concomitant ASMs. Given the lack of evidence currently available on PK behavior of CNB, this study offers valuable information that could be useful for the tailored therapy of seizures. However, these findings are exploratory and hypothesis-generating, and they must be confirmed in bigger, prospective, multicenter investigations.

## Figures and Tables

**Table 1:** Demographic and clinical characteristics of enrolled individuals.

	Drug-Resistant Focal Epilepsy n= 17
Sex, n (female %)	9 (52.9%)
Age, years	52 ± 14
Age at onset, years	25 ± 22
Epilepsy duration, years	27 ± 23
Weight (kg)	75 ± 16
BMI	25.44 ±4.34

**Table 2:** PK parameters (AUC, ΔC/ΔD and C/D ratio) and clinical-anthropometric features of the enrolled people with epilepsy treated with add-on CNB. Participants were stratified into low-, medium- and high-exposure clusters based on AUC values.

Patient ID	AUC	Max_ Slope	C/D_ 200	Weight (kg)	BMI	Sex	Age (Year)	Age Onset (Year)	Epilepsy Duration (Year)	Seizures/Month	Age Cluster (Year)	Cluster_AUC
1	1508	0.010	0.082	80	27	F	68	10	58	30	>60	Medium
2	2444	0.112	0.123	70	29	F	70	63	7	1	>60	High
3	1134	0.073	0.059	80	30	F	54	38	16	4	40–60	Low
4	988	0.034	0.043	80	26	M	53	52	2	2	40–60	Low
5	1523	0.080	0.076	84	23	M	30	20	4	1	<40	Medium
6	1612	0.042	0.078	57	19	F	45	42	3	3	40–60	Medium
7	1888	0.282	0.132	90	31	M	21	13	8	10	<40	Medium
8	745	0.051	0.045	80	24	M	40	12	30	1	40–60	Low
9	2405	0.031	0.094	54	21	F	53	4	49	2	40–60	High
10	3754	1.104	0.384	56	22	F	56	10	47	4	40–60	High
11	1260	0.124	0.078	105	35	M	64	2	62	300	>60	Low
12	1262	0.081	0.092	75	27	M	56	55	1	30	40–60	Low
13	2358	0.013	0.076	52	18	F	54	5	50	12	40–60	High
14	1767	0.354	0.150	92	25	M	67	6	61	9	>60	Medium
15	2109	0.293	0.145	57	23	F	62	55	7	2	>60	Medium
16	2715	0.133	0.134	99	29	F	31	1	33	5	<40	High
17	2136	0.155	0.090	70	23	M	57	44	14	1	40–60	Medium

**Table 3:** Distribution of sex across AUC exposure clusters.

	Female	Male
High	5	0
Low	1	4
Medium	3	4

**Table 4:** Univariate ANOVA of concomitant antiseizure medications and CNB plasmatic concentration.

*Drug*	*F-Value*	*p-Value*	*Significance*
*LEV*	2.615	0.127	No
*PER*	0.191	0.669	No
*VPA*	2.451	0.138	No
*ESL*	0.003	0.955	No
*LTG*	0.036	0.852	No
*CBZ*	1.217	0.287	No
*LCM*	5.478	0.0335 *	Yes
*BRV*	0.373	0.550	No
*PB*	1.056	0.320	No
*OXC*	0.673	0.425	No
*GBP*	0.459	0.508	No
*CLB*	1.437	0.249	No
*CBD*	1.437	0.249	No
*TPM*	11.250	0.0044 **	Yes

* Statistically significant (*p* < 0.05). ** Statistically significant (*p* < 0.01)

## Data Availability

The raw data supporting the conclusions of this article will be made available by the authors on request. The data is available by contacting the corresponding author.

## Supplementary Materials

The following supporting information can be downloaded at: https://www.mdpi.com/article/10.3390/pharmaceutics18010092/s1, Table S1: FDR effect sizes, Exploratory statistical analyses of the association between cenobamate plasma exposure and clinical-demographic variables and concomitant antiseizure medications. Unadjusted and false discovery rate (FDR)–corrected *p*-values together with effect sizes (Cohen’s d or Spearman’s ρ) are reported.

## Abbreviations

The following abbreviations are used in this manuscript:

CNB	Cenobamate
ASM	Anti-seizure medication
CBZ	Carbamazepine
CLB	Clobazam
BRV	Brivaracetam
VPA	Valproic Acid
LCM	Lacosamide
TPM	Topiramate
LEV	Levetiracetam
PER	Perampanel
ESL	Eslicarbazepine
LTG	Lamotrigine
PHT	Phenytoin
PB	Phenobarbital
OXC	Oxcarbazepine
GBP	Gabapentin
CBD	Cannabidiol
UHPLC-MS/MS	Ultra-High-Performance Liquid Chromatography—Tandem Mass Spectrometry
LC-MS/MS	Liquid Chromatography—Tandem Mass Spectrometry
UPLC	Ultra-Performance Liquid Chromatography
IS	Internal Standard
EDTA	Ethylenediaminetetraacetic acid
